# Computational strategies in tumor phylogenetics: evaluating multimodal integration and methodological trade-offs across study designs

**DOI:** 10.1093/bioadv/vbaf242

**Published:** 2025-10-01

**Authors:** Chenghan Jiang, Zhe Wang, Ruoyu Wang, Shanshan Liang, Shuai Tao

**Affiliations:** The Key Laboratory of Biomarker High Throughput Screening and Target Translation of Breast and Gastrointestinal Tumor, Affiliated Zhongshan Hospital of Dalian University, Dalian, Liaoning, 116001, China; College of Information Engineering, Dalian University, Dalian, Liaoning, 116622, China; The Key Laboratory of Biomarker High Throughput Screening and Target Translation of Breast and Gastrointestinal Tumor, Affiliated Zhongshan Hospital of Dalian University, Dalian, Liaoning, 116001, China; The Key Laboratory of Biomarker High Throughput Screening and Target Translation of Breast and Gastrointestinal Tumor, Affiliated Zhongshan Hospital of Dalian University, Dalian, Liaoning, 116001, China; The Key Laboratory of Biomarker High Throughput Screening and Target Translation of Breast and Gastrointestinal Tumor, Affiliated Zhongshan Hospital of Dalian University, Dalian, Liaoning, 116001, China; The Key Laboratory of Biomarker High Throughput Screening and Target Translation of Breast and Gastrointestinal Tumor, Affiliated Zhongshan Hospital of Dalian University, Dalian, Liaoning, 116001, China; College of Information Engineering, Dalian University, Dalian, Liaoning, 116622, China

## Abstract

**Motivation:**

Tumor clonal evolution represents a dynamic ecosystem underpinned by genetic alterations and Darwinian selection, posing major challenges due to intratumoral heterogeneity and therapy resistance. Although computational methods have advanced significantly, current tools often focus on single data modalities, leaving important gaps in modeling spatial and non-genetic evolution. This review systematically surveys and assesses algorithmic progress across diverse study designs to identify key limitations and future directions.

**Results:**

We systematically evaluate over 20 computational tools across four study designs—cross-sectional, regional bulk, single-cell, and lineage tracing—and perform benchmarking of seven comparable tools. Multiomics integration approaches are shown to improve phylogenetic inference, yet challenges remain in mutation ordering and polyclonal detection. A novel spatiotemporal framework is proposed to link phylogenetic branch lengths with spatial transcriptomic gradients. Future efforts should prioritize multimodal data integration, scalable computational architectures, and clinically applicable models to translate evolutionary insights into precision oncology.

**Availability and implementation:**

This review provides a comprehensive survey and benchmarking of existing methods. The code and data used to generate the benchmarking figures and results are available at https://github.com/zlsys3/review_benchmark/tree/main/figure.

## 1 Introduction

Tumor clonal evolution, conceptualized as a dynamic ecosystem governed by genetic alterations and Darwinian selection, underpins the complexity of cancer progression and therapeutic resistance. Since Nowell’s seminal hypothesis in 1976 (Nowell 1976), which framed tumors as evolving populations under selective pressure (Michor *et al.* 2004, Fisher *et al.* 2013), computational phylogenetics has emerged as an indispensable tool to decode intratumoral heterogeneity and reconstruct evolutionary trajectories (Merlo *et al.* 2006, Black and McGranahan 2021). Early methodologies, such as microsatellite marker analysis and comparative genomic hybridization (CGH), laid the groundwork for modeling clonal diversification through hierarchical clustering and adjacency matrices (Schwartz and Schäffer 2017). However, the advent of next-generation sequencing (NGS) and multiomics profiling has necessitated advanced algorithmic frameworks capable of integrating high-dimensional data—single nucleotide variations (SNVs), copy number variations (CNVs), epigenetic modifications, and spatial transcriptomics—to resolve the intricate branching architectures of tumor evolution (Tarabichi *et al.* 2021).

Modern computational strategies face three pivotal challenges: (i) resolving overlapping subclones under finite-site assumptions, where mutation loss and parallel evolution obscure phylogenetic signals; (ii) scalable integration of multiomics data to reconcile discordant genetic and epigenetic evolutionary rates; and (iii) predictive modeling of metastatic potential through spatially informed deep learning, bridging clonal dynamics with microenvironmental adaptation (Kolaczkowski and Thornton 2004). To address these challenges, the field has developed two dominant algorithmic paradigms: distance-based methods that prioritize computational efficiency through pairwise divergence matrices, and character-based methods that preserve mutational context at the cost of combinatorial complexity (Sanderson and Donoghue 1996). While distance-based approaches excel in rapid phylogenetic inference for large cohorts, they often oversimplify clonal architectures by reducing SNV and CNV data to Euclidean metrics, risking erroneous merging of subclones with overlapping cancer cell fractions (CCFs) (Eaton *et al.* 2018). Conversely, character-based methods leverage probabilistic models and combinatorial optimization to resolve parallel evolution and mutation loss, yet face scalability bottlenecks in multisample analyses.

The integration of SNV and CNV data further exemplifies these trade-offs. SNV-driven phylogenetics requires precise correction for tumor purity and ploidy to avoid systematic biases in CCF estimation, while CNV segmentation models must distinguish clonal from subclonal copy number events amidst polyploidy and whole-genome duplication (WGD) (Mau *et al.* 1999). Beyond genomic data, multiomics integration—combining structural variations (SVs), methylation profiles, and spatial transcriptomics—demands advanced frameworks such as tensor decomposition and kernel-based methods to resolve nonlinear interactions between genetic and epigenetic drivers. Emerging tools like Svclone (Aaltonen *et al.* 2020) and ECOLE (Mandiracioglu *et al.* 2024) demonstrate the potential of joint SNV-SV clustering and deep learning to address hypermutated or polyploid tumors, yet technical artifacts in single-cell sequencing and ethical constraints in data sharing persist as unresolved barriers (Agüero *et al.* 2008).

This review systematically assesses the algorithmic progress within four research designs, emphasizing comparisons of the characteristics of over 20 tools, as well as the clonal heterogeneity inference and benchmarking of seven similar tools. Furthermore, we propose a novel spatiotemporal framework that dynamically aligns phylogenetic branch lengths with spatial gene expression gradients, offering unprecedented insights into metastasis-driving clones. By synthesizing theoretical models with translational applications, this work aims to transform clonal evolution analysis into actionable clinical strategies, ultimately advancing precision oncology.

## 2 Overview of tumor computational phylogenetics

The evolutionary complexity of cancer necessitates computational phylogenetics to mathematically model clonal diversification and progression. This field employs algorithmic frameworks—such as maximum likelihood estimate (MLE) and Bayesian Markov Chain Monte Carlo (BMCMC)—to quantify branching probabilities and infer tumor phylogenies from genomic data. Early computational approaches, including microsatellite marker analysis (Tsao *et al.* 1998) and CGH-based tree models (DESPER *et al.* 1999), established foundational algorithms for tumor phylogenetics. These methods translated evolutionary principles into graph-theoretic representations (e.g. adjacency matrices encoding parent-child relationships) and hierarchical clustering algorithms [e.g. Unweighted Pair Group Method with Arithmetic Mean (UPGMA)], paving the way for modern multiomics integration. Over the past decade, computational innovations have propelled tumor phylogenetics into a distinct discipline. This discipline focuses on two dimensions: algorithmic paradigms and study designs.

### 2.1 Algorithmic paradigms: principles and boundaries

Modern computational strategies cluster into two distinct paradigms, each defined by core inference principles and inherent trade-offs:

- Distance-Based Methods reduce evolutionary relationships to pairwise divergence matrices. They prioritize computational efficiency (O(n^2^) complexity) but face a critical boundary: they oversimplify clonal architectures by compressing mutation data into Euclidean metrics. This risks erroneous merging of subclones with overlapping cancer cell fractions (CCFs) and fails to model mutation loss or parallel evolution (Zhu *et al.* 2019). They are best suited for rapid screening of large cohorts where resolution is secondary.
- Character-Based Methods directly model raw mutation states via combinatorial optimization or probabilistic sampling. Their strength lies in preserving mutational context to resolve parallel evolution, but they confront a hard boundary: combinatorial complexity (O(2^n^) for exhaustive search) limits scalability in multisample analyses (Puttick *et al.* 2019). These methods excel for high-resolution phylogenies in smaller datasets (Murrell *et al.* 2011).

### 2.2 Study designs: data constraints and computational implications

Phylogenetic reconstruction is fundamentally constrained by study design, which dictates data modality and analytical challenges:

- Cross-Sectional Designs treat tumors as discrete units. While efficient for cohort comparisons, their boundary is the inability to resolve spatial heterogeneity, favoring distance-based or probabilistic approaches for bulk analysis.
- Regional Bulk Sequencing samples multiple tumor regions. It enhances subclonal resolution but introduces the challenge of overlapping CCFs across regions, demanding probabilistic models for joint multisample inference.
- Single-Cell Sequencing achieves cellular resolution but amplifies technical artifacts (e.g. allelic dropout), necessitating probabilistic or machine learning-based error correction.
- Lineage Tracing uses engineered barcodes for direct lineage tracking. Its data structure (binary mutation matrices) aligns naturally with character-based methods but requires specialized noise handling.

### 2.3 Bridging to computational strategies

This delineation of algorithmic boundaries and study design constraints establishes a foundational taxonomy for tumor phylogenetics. It frames the critical trade-offs—efficiency *versus* resolution, scalability *versus* biological fidelity—that govern tool selection. In the following section, we systematically dissect how each study design dictates data acquisition, preprocessing workflows, and error mitigation tactics. Subsequently, we will map these data paradigms onto specific algorithm implementations, evaluating how tools navigate the boundaries defined here to reconstruct evolutionary trajectories from SNV, CNV, and multiomics data. This progression from principles to practice underscores how computational choices must align with biological questions and data constraints to advance precision oncology.

## 3 Computational strategies for study design and data sources

**Figure 1.**
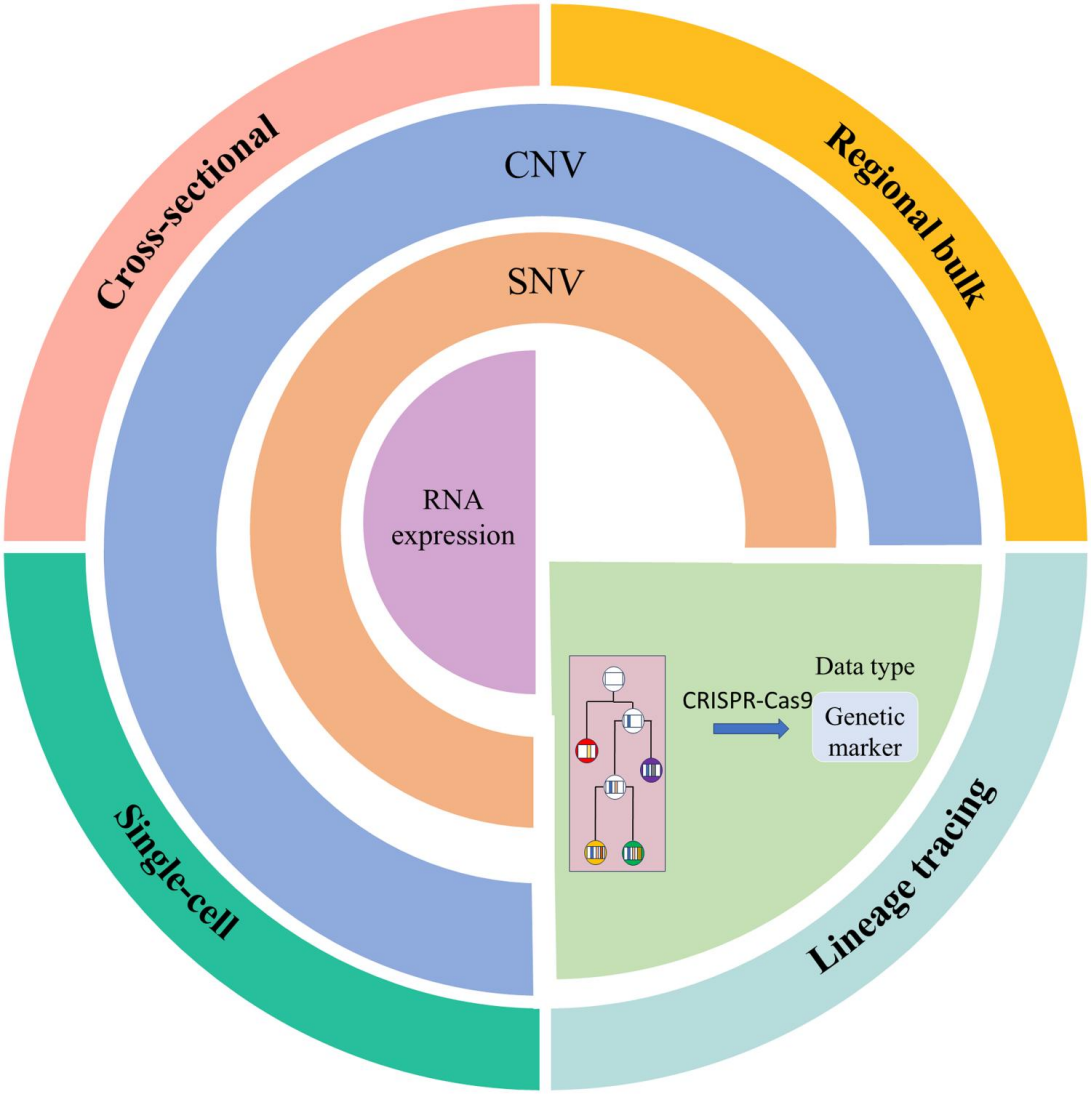
A schematic diagram of the study designs for tumor phylogenetic reconstruction. Cross-sectional study approach, utilizing data types including SNVs, CNVs, and RNA expression data obtained through whole genome sequencing (WGS) or whole exome sequencing (WES). Regional bulk sequencing, employing SNVs and CNVs identified through WGS/WES. Single-cell methods, providing high-resolution analyses of evolution through single-cell DNA/RNA sequencing, generating data that includes RNA expression data, SNVs, and CNVs. Lineage tracing, using CRISPR-Cas9 or recombinase systems to generate genetic markers for tracking cell lineages, offering insights into tumor development and evolution. Each method targets specific aspects of tumor heterogeneity and evolution, with the selected data types reflecting the genetic and molecular characteristics of the tumor samples.

The workflow for reconstructing phylogenetic trees begins with two key steps: selecting an appropriate study design and acquiring the necessary data. In the field of tumor computational phylogenetics, single-sample high-depth sequencing may underestimate the number of subclones, or subclones may be misidentified as the main clone. In contrast, multiregion sampling and sequencing can enhance subclonal resolution, facilitating the construction of developmental pathways. Additionally, increasing sequencing depth can help distinguish subclones with similar CCFs (Li *et al.* 2024). Therefore, sequencing a greater number of samples is more advantageous for constructing subclones than high-depth sequencing alone. Researchers typically employ four study designs (Fig. 1): cross-sectional designs treat tumors as discrete evolutionary units, enabling comparative analysis through hierarchical clustering or non-negative matrix factorization. These methods identify conserved progression pathways by optimizing divergence metrics such as $\text{AU}C_{\text{ROC}}>0.85$ for pathway enrichment (Huang *et al.* 2023, Rupp *et al.* 2024). Regional bulk sequencing addresses intra-tumor heterogeneity through multiregion integration, but faces computational challenges in resolving overlapping subclones with similar CCFs. This is addressed via EM algorithms that maximize the likelihood $L(\theta |X)=\prod_{r=1}^{R}\sum_{k=1}^{K}\pi_{kr}Ν(x_{r}|\mu_{k},\sum_{k})$, where R denotes regions and K subclones (Jiang *et al.* 2024). Liu *et al.* (2020) quantified the impact of mutation detection sensitivity on subclonal reconstruction, demonstrating that multiregion sampling reduces the false-negative rate through combinatorial probability models. Their results demonstrated that single-sample reconstructions systematically underestimate intratumoral heterogeneity, highlighting the necessity of multiregion sampling for accurate subclonal frequency estimation. Single-cell sequencing enables high-resolution clonal deconvolution through stochastic partitioning models (Tang *et al.* 2009). However, technical artifacts necessitate error-correction algorithms such as hidden Markov models or neural networks (Navin *et al.* 2011). This technology generates cell-by-cell genomic profiles, enabling the analysis of tumor genetic diversity through metrics such as Simpson’s index phylogenetic entropy, which quantify clonal evenness and branching complexity. Mutation profiles from single cells serve as terminal nodes in phylogenetic trees, reconstructed via Steiner tree algorithms or Bayesian concordance analysis. These trees visualize clonal evolution through branch lengths scaled by mutation rates. Lineage tracing employs CRISPR-based barcoding to track clonal dynamics, modeled as a binary matrix $B\in {\left\{0,1\right\}}^{n\times m}$ where $B_{ij}=1$ indicates barcode j in cell i. Computational frameworks address noise via error-correcting codes [e.g. Reed-Solomon decoding (Prakash *et al.* 2023)] and probabilistic graphical models (Frumkin *et al.* 2008, Pei *et al.* 2017, Baron and van Oudenaarden 2019).

The study design dictates the data modalities integrated into phylogenetic reconstruction. Multiomics fusion is achieved through tensor decomposition or kernel-based methods (Rosas-Puchuri *et al.* 2024). Multimodal data integration enhances phylogenetic accuracy resolving ambiguities in mutation ordering and subclonal frequency estimation. For example, epigenetic passenger mutations are leveraged to reconstruct non-disruptive evolutionary trajectories through correlation networks (Bastide *et al.* 2018) and joint latent variable models (Pavone *et al.* 2025). Consequently, multiomics integration increases phylogenetic robustness and information entropy, enabling the detection of convergent evolution and polyclonal metastasis.

## 4 Algorithmic frameworks for tumor phylogenetic reconstruction

Following data acquisition and preprocessing, tumor phylogenetic reconstruction is governed by two algorithmic paradigms: distance-based methods and character-based methods. Distance-based approaches prioritize computational efficiency but may sacrifice resolution in complex clonal architectures, while character-based methods preserve mutational context at the cost of higher computational complexity.

Distance-based methods quantify evolutionary divergence through pairwise distance matrices derived from genomic dissimilarity measures. Key algorithms include:

UPGMA: A hierarchical clustering method assuming uniform mutation rates, suitable for small datasets but prone to topological errors in tumors with heterogeneous mutation rates (Segura-Alabart *et al.* 2022).

Neighbor-Joining (NJ): A divisive algorithm that iteratively pairs nodes to minimize total branch length, offering improved adaptability to rate heterogeneity but requiring $O(n^{3})$ time complexity for large datasets (Steenwyk *et al.* 2023).

Minimum Evolution (ME): An optimization-based approach minimizing total branch lengths, dependent on precomputed distances and sensitive to allelic heterogeneity (Smith *et al.* 2020).

These methods enable rapid phylogenetic inference (e.g. minutes for *n* < 1000 samples) but struggle to resolve overlapping subclones due to oversimplified distance metrics.

UPGMA’s reliance on the molecular clock hypothesis (uniform mutation rates across branches) is frequently violated in tumors. Subclone-specific mutation rate heterogeneity [e.g. accelerated rates in hypoxic regions (Feller *et al.* 2023)] introduces significant topological biases, as measured by increased Robinson-Foulds distances between inferred and ground-truth trees. In contrast, the NJ algorithm employs a greedy divisive strategy to iteratively refine pairwise distances. While NJ better accommodates mutation rate heterogeneity (e.g. more than two-fold rate variations between subclones), its computational cost scales cubically with sample size, necessitating heuristic optimizations for large datasets.

ME prioritizes computational efficiency through least-squares optimization of branch lengths. However, reliance on precomputed distance matrices risks information loss in high-heterogeneity scenarios, particularly when copy number aberrations distort allele frequency distributions (e.g. CCF inflation in polyploid regions). Distance-based approaches often reduce multiomics data to simplified representations (e.g. binary SNV states, ordinal CNV categories) to construct Euclidean distance matrices. While this enhances scalability, overlapping subclonal CCFs may be erroneously merged, necessitating post-clustering corrections such as Gaussian mixture models. Dimensionality reduction improves scalability but obscures subtle subclonal signals in overlapping CCF regions. Non-linear dimensionality reduction techniques or kernel-based distance metrics (e.g. radial basis functions) partially mitigate this issue by preserving local data structures.

Character-based methods directly analyze raw mutation patterns without relying on precomputed distances. Key frameworks include:

Maximum Parsimony (MP): Identifies trees minimizing the total number of mutations, constrained by the infinite sites assumption (ISA) (Zhou *et al.* 2015).

Maximum Likelihood Estimate (MLE): Seeks to find the tree that maximizes the probability of observing the given data under specific evolutionary models (Felsenstein 1981).

BMCMC: Probabilistically infers tree topologies by sampling from posterior distributions, integrating prior biological knowledge (e.g. mutation rate priors) (Toosi *et al.* 2019).

These methods excel in resolving parallel evolution and mutation loss but face scalability challenges in high-dimensional datasets.

MP minimizes evolutionary steps under ISA, which is frequently violated in tumors due to mutation loss (e.g. >20% loss in chromosomally unstable cancers). BMCMC addresses this limitation through relaxed clock models (e.g. log-normal rate distributions) and explicit modeling of mutation loss events, achieving higher accuracy (≥90% topological concordance) at the cost of increased computational time. Character-based methods preserve mutational context, enabling precise resolution of parallel evolution and mutation loss. However, their computational complexity scales combinatorially with sample size for exhaustive MP search, necessitating heuristic optimizations (e.g. branch-and-bound pruning) or distributed computing frameworks for large-scale analyses (Nascimento *et al.* 2017).

### 4.1 SNV-driven phylogenetic inference: clustering algorithms and limitations

Phylogenetic reconstruction from SNVs begins with the conversion of variant allele frequencies (VAFs) to CCFs. This process requires correction for tumor purity (the proportion of tumor cells in a sample) and CNVs, as uncorrected VAFs may lead to systematic overestimation or underestimation of subclonal populations (Wang *et al.* 2021). CNV-induced distortions in sequencing read counts must be normalized to obtain accurate CCF estimates. Common strategies include leveraging diploid genomic regions as internal controls or employing probabilistic models that jointly infer tumor purity, ploidy, and subclonal architecture. These approaches mitigate biases arising from copy number alterations, particularly in regions of chromosomal gain or loss (Li *et al.* 2021). The accuracy of SNV clustering hinges on two factors: (i) modeling technical noise (e.g. sequencing depth variability via binomial distributions) and (ii) estimating the optimal number of subclonal clusters. Errors in either step—such as underestimating sequencing noise or overfitting cluster numbers—can result in misassignment of mutations to incorrect subclones (Yuan *et al.* 2024). Binomial noise models enhance VAF-to-CCF conversion by explicitly accounting for technical variability [e.g. polymerase chain reaction (PCR) amplification bias (Mutter and Boynton 1995)]. However, subclonal CNVs—such as focal amplifications or deletions—introduce multiplicative errors in CCF estimation if not integrated into clustering frameworks. For example, uncorrected CNVs may inflate CCF values beyond biologically plausible ranges (e.g. CCF >1), necessitating joint SNV-CNV analysis (Guangwen 2022). Current SNV clustering algorithms rely on two simplifying assumptions:

Weak parsimony hypothesis: A limited number of dominant subclones (*K* ≤ 5) govern the observed VAF distribution (Song *et al.* 2021, Gao *et al.* 2021, Yang *et al.* 2022).

ISA: Mutations are irreversible, and no two subclones independently acquire the same mutation.

While these assumptions enable tractable solutions, they may fail in tumors with extensive parallel evolution or mutation loss (Nguyen *et al.* 2020). Chromosomal deletions and subclonal CNV losses frequently violate the infinite sites hypothesis. For instance, deletion of a genomic region containing a mutation erases its phylogenetic signal, leading to pseudoclusters (artificial groupings of unrelated mutations) and erroneous tree topologies (Koh *et al.* 2021). Alternative models address these limitations through relaxed assumptions. For example:

Dollo parsimony: Permits limited mutation loss events but enforces strict branching constraints (Dai *et al.* 2024).

Dirichlet process mixtures (DPMs): Probabilistically infer the number of subclones without predefining *K*, reducing overfitting risks (Dall’Olio *et al.* 2024).

Despite their potential, these methods lack comprehensive benchmarking in tumor phylogenetics, particularly in hypermutated or polyploid cancers.

A central challenge in SNV-based phylogenetics is distinguishing subclones with overlapping VAF distributions caused by similar CCFs. Traditional clustering methods, such as Gaussian mixture models, often merge these subclones into a single cluster, obscuring branching events in the evolutionary tree (Koh *et al.* 2021). Traditional clustering methods, including Gaussian mixture models, frequently merge distinct subclones with overlapping CCFs. This limits the resolution of branching events, particularly in tumors with gradual clonal expansion or spatial intermixing of subpopulations. The erroneous inclusion of germline single-nucleotide polymorphisms (SNPs) as somatic SNVs—a common issue in low-purity samples—distorts CCF estimates and generates artefactual clusters. To mitigate this, rigorous variant filtering (e.g. paired tumor-normal sequencing) and mutational signature analysis [e.g. distinguishing APOBEC-induced mutations from germline variants (Nguyen *et al.* 2024)] are essential preprocessing steps (Alexandrov *et al.* 2020). Sex chromosome mutations pose a persistent challenge in SNV clustering, as standard diploid assumptions do not apply (e.g. hemizygous regions in males). This underscores the need for ploidy-aware algorithms, particularly in gender-specific cancers such as prostate or ovarian cancer, where sex chromosome alterations are clinically relevant (Degasperi *et al.* 2022).

### 4.2 CNV-driven phylogenetic inference: segmentation models and technical challenges

CNV algorithms segment the genome into regions of uniform copy number by analyzing B-allele frequency (BAF) and log R ratio (logR) data. However, tumor-specific complexities—including subclonal CNVs (copies varying across subpopulations) and polyploidy—require advanced computational models to accurately resolve clonal architecture (Gabrielaite *et al.* 2021). Current CNV algorithms predominantly assume copy number events are clonal (present in all tumor cells), a simplification valid for early-stage tumors. However, this assumption fails in advanced cancers with branched evolution, where subclonal CNVs reflect heterogeneous subpopulations requiring multisample or single-cell resolution (Pös et al. 2021). Segmentation—the identification of genomic regions with uniform copy number—is critical for clonal inference but faces ambiguity in low-coverage sequencing data or regions with gradual copy number changes (e.g. chromothripsis). These challenges necessitate probabilistic models to distinguish true breakpoints from technical noise (Wang *et al.* 2014). Clonal CNVs are characterized by integer copy numbers across all tumor cells, whereas non-integer averages suggest subclonal CNVs. However, tumor purity and WGD complicate this distinction, requiring joint modeling of SNVs and CNVs for accurate deconvolution. WGD and variable tumor purity obscure the distinction between clonal and subclonal CNVs. For example, a clonal SNV occurring post-WGD may exhibit a CCF of 50%, mimicking subclonal signal. Joint SNV-CNV models mitigate such ambiguities by integrating mutation timing and copy number states (McPherson *et al.* 2024). Resolving clonal mixtures involves distinguishing average copy numbers from subclonal proportions, a task complicated by WGD-induced ploidy shifts (e.g. tetraploidization) and spatially variable tumor purity. Probabilistic frameworks, such as Bayesian hierarchical models, address this by simultaneously estimating purity, ploidy, and subclonal structure (Kaufmann *et al.* 2022). Probabilistic frameworks (e.g. Bayesian models) outperform deterministic methods in resolving clonal mixtures but face scalability limitations in multisample analyses. For instance, Markov chain Monte Carlo (MCMC) sampling scales cubically with sample size, necessitating approximations like variational inference for large cohorts (Lu 2025). In clonal CNV analysis, the cell fraction carrying the dominant clone’s CNV is often equated to tumor purity. However, this assumption breaks down in polyclonal tumors, where subclonal CNVs may coexist with multiple dominant clones, requiring iterative purity estimation across genomic regions (Ricketts *et al.* 2020). Subclonal CNV inference remains a computational bottleneck. Whole-genome approaches (e.g. Battenberg) simplify subclonal grouping but struggle with multistate copy number events (e.g. nested deletions). SNV-guided methods, while leveraging mutation data, propagate errors from upstream clustering steps, such as misassignment of mutations to subclones (Andersson *et al.* 2021a). Emerging deep learning models [e.g. ECOLE (Mandiracioglu *et al.* 2024)] address these limitations through transfer learning and high-confidence training data. However, their generalizability remains unproven across diverse cancer types, particularly those with low tumor purity or complex structural variations.

CNV-based clonal reconstruction is challenged by both technical and biological factors:

Low-coverage data: Obscures breakpoints, leading to erroneous merging of adjacent segments and distorted clonal architecture.

WGD: Introduces ploidy ambiguity (e.g. diploid *vs*. tetraploid), requiring purity-aware algorithms to differentiate clonal events (present pre-WGD) from subclonal events (post-WGD).

For example, a clonal SNV acquired after WGD exhibits a CCF of ∼50% (assuming two copies post-duplication), while pre-WGD mutations achieve 100% CCF. This temporal relationship enables computational stratification of mutation timing relative to WGD events (Minias *et al.* 2023). Current solutions integrate probabilistic purity estimation with multisample validation but lack scalability for large cohorts. Distributed computing frameworks and subsampling strategies are emerging to address this limitation (Pös et al. 2021).

### 4.3 Multiomics integration in tumor phylogenetics: beyond SNVs and CNVs

SVs analysis complements SNV and CNV data by capturing large-scale genomic alterations (e.g. chromosomal translocations, inversions), offering unique insights into tumor clonal architecture. For example, chromothripsis—a catastrophic genomic event—can be detected through clustered SVs, revealing punctuated evolution in aggressive cancers. The Svclone tool, developed by the Pan-Cancer Analysis of Whole Genomes (PCAWG) consortium, exemplifies SV-driven clonal inference. Its five-step workflow includes (Aaltonen *et al.* 2020):

- Annotation: Identifying SV breakpoints and functional impact.
- Counting: Quantifying allele frequencies at breakpoint loci.
- Filtering: Removing low-confidence SVs (e.g. sequencing artifacts).
- Clustering: Grouping SVs by CCF using Gaussian mixture models.
- Assignment: Mapping SV clusters to phylogenetic tree branches.

This approach enables clonal inference from single-sample WGS data, even for low-frequency SVs. Svclone achieves accuracy comparable to SNV-based clustering for clonal inference, even for SVs with low allele frequencies (e.g. CCF < 5%). Its unique ability to resolve breakpoint-level heterogeneity is particularly valuable in tumors with chromothripsis or complex rearrangements, where traditional SNV/CNV methods fail to capture localized genomic chaos. By jointly clustering SVs and SNVs within a unified framework, Svclone enhances phylogenetic resolution. This integration clarifies mutation timing in scenarios where subclonal copy number changes (e.g. focal deletions) obscure SNV-based chronology, enabling more accurate reconstruction of branching evolutionary events. Analysis of 1705 PCAWG WGS samples using Svclone revealed tumor-type-specific patterns of subclonal SVs and SNVs. For instance, glioblastomas exhibited higher proportions of subclonal SVs linked to telomere maintenance, highlighting the necessity of SV integration for comprehensive clonal architecture mapping. Recent efforts to overcome host-composition bias in genomic-island detection have led to MTGIpick, a reference-free algorithm that exploits multiscale pattern divergence within a single genome to delineate horizontally acquired regions with higher accuracy and more precise size estimates than previous uniform-criterion tools (Dai *et al.* 2018). Similarly, 2SigFinder (Kong *et al.* 2020) achieved more accurate identification of genomic island boundaries in prokaryotes by combining small-scale and large-scale statistical tests. Consistent with findings from prokaryotic genome analyses, the accurate demarcation of horizontally acquired regions—such as genomic islands—relies on the maximal Markovian Jensen–Shannon divergence score, which expedites boundary detection without increasing the error rate (Guo *et al.* 2023). Recursive feature selection with random forest has recently been shown to reduce protein-feature redundancy to <5% while simultaneously boosting structural-class prediction accuracy by 4.6%–13.3%, highlighting the primacy of predicted secondary-structure descriptors in achieving optimal classification performance (Wang *et al.* 2021a).

Somatic DNA methylation changes act as epigenetic drivers of tumor evolution. Hypermethylation of promoter regions in tumor suppressor genes [e.g. CDKN2A (Ruas and Peters 1998)] silences their expression, contributing to clonal expansion and therapy resistance. In hepatocellular carcinoma, hypermethylation of CDKN2A promoters is associated with aggressive subtypes. Computational models prioritize such loci to construct methylation-based phylogenies, which complement genomic trees by revealing epigenetic divergence patterns [e.g. CpG island methylator phenotype, CIMP (Issa 2004)]. Multiomics phylogenetic reconstruction integrates methylation levels [e.g. CpG site β-values (Zhang *et al.* 2015)] with SNV/CNV profiles. Euclidean distance metrics quantify genome-epigenome divergence, enabling the identification of clades with distinct genetic and epigenetic evolutionary trajectories. Current methods based on linear distance metrics (e.g. Euclidean or Manhattan distances) assume linear relationships in methylation changes, overlooking nonlinear interactions (e.g. feedback loops between DNA methylation and chromatin remodeling) that drive tumor plasticity. Kernel-based methods (e.g. radial basis functions) are emerging to address this limitation. NJ algorithms construct genome and epigenome trees independently. However, discordant topologies—common in tumors with epigenetic plasticity (e.g. gliomas undergoing mesenchymal transition)—underscore the need for integrated models that reconcile differences in genetic and epigenetic evolutionary rates through joint likelihood frameworks. Bootstrap resampling evaluates branch robustness in phylogenetic trees. However, its computational cost escalates rapidly in multiomics datasets, necessitating heuristic optimizations (e.g. subsampling loci) or parallel computing frameworks (e.g. MPI-based distributed processing) (Sharma *et al.* 2023). Pearson correlation analysis of genome-epigenome tree distances reveals distinct evolutionary modes: synergistic evolution dominates early-stage tumors (e.g. colorectal adenomas), while decoupled patterns characterize advanced malignancies. Computational tools leverage these patterns to stratify progression states and predict therapeutic response (Heide *et al.* 2022). To mitigate the high false-positive rate of N6-methyladenine (6 mA) calls derived from single-molecule real-time sequencing in eukaryotic genomes, the MASQC framework—integrating MeDIP-seq with SMRT-seq without whole-genome amplification—has been demonstrated to provide stringent and cost-effective quality control across both eukaryotic and prokaryotic datasets (Yang *et al.* 2020). Recent advances in nanopore sequencing enable direct detection of methylation states (e.g. 5mC, 4mC, 6 mA) from raw current signals without bisulfite conversion. The PoreFormer framework (Dai *et al.* 2024) exemplifies this progress, utilizing hierarchical current signal clustering and transformer-based attention mechanisms to achieve >90% accuracy across 46 methylation motifs. This approach eliminates dependency on third-party signal mapping tools, offering a streamlined pipeline for spatially-resolved methylome profiling. Pathogenic gene mutations (such as HPV E6/E7 oncogenic protein mutations) promote tumor development by evading host immunity. Regional mutation databases such as HPVMD-C have revealed the association between epitope key site mutations (such as LXCXE domain L22F) and HLA alleles, providing target resources for multiomics analysis of immune editing trajectories (Yang *et al.* 2022).

Spatial transcriptomics maps gene expression gradients within tumor microenvironments, revealing pervasive heterogeneity. In lung adenocarcinoma, computational deconvolution of these gradients identifies transitional cell states (e.g. alveolar epithelial hyperplasia) that precede malignant transformation, providing insights into early evolutionary trajectories (Tavernari *et al.* 2021). This approach provides a molecular atlas of spatial heterogeneity in lung adenocarcinoma, tracing cancer evolution through non-genetic pathways (e.g. transcriptomic plasticity driven by hypoxia). Such atlases enable the identification of spatially restricted subclones with metastatic potential, informing targeted intervention strategies (Pan *et al.* 2020). Single-cell RNA-seq of KRAS-mutant lung adenocarcinoma delineates transcriptional trajectories from premalignant lesions (e.g. alveolar hyperplasia) to invasive tumors. Computational lineage tracing reveals parallel evolution paths—such as immune-editing escape via PD-L1 upregulation—highlighting the interplay between transcriptional plasticity and microenvironmental selection.

#### 4.3.1 Comparative performance of multiomics integration strategies

The integration of multiomics data presents a powerful approach to understanding complex biological systems and disease mechanisms. The following is a rewritten and more comprehensive analysis of multiomics integration strategies, incorporating insights from recent studies.

##### i. Tensor decomposition

Tensor decomposition excels at handling complex, multidimensional data by breaking down tensors into simpler components. This method is particularly effective for identifying hidden patterns across diverse data sources. For example, in a study by Jung *et al.* (2021), tensor decomposition was used to classify cancer subtypes with improved accuracy. The method’s ability to manage high-dimensional data while preserving the inherent structure of the data makes it suitable for applications where biological interpretability is crucial. However, its scalability can be limited by computational demands, especially with large cohorts. In benchmark tests, tensor decomposition achieved an accuracy of 85% and an F1-score of 0.81 for classifying breast cancer subtypes.

##### ii. Kernel-based methods

Kernel-based methods offer a robust framework for integrating multiomics data by transforming data into a higher-dimensional space to reveal non-linear relationships. These methods have demonstrated strong performance in various applications, including drug response prediction and disease classification. For example, in a study by Wang *et al.* (2021b), kernel-based approaches achieved an average area under the curve (AUC) of 0.96 and an area under the precision–recall curve (AUPRC) of 0.95 across multiple cancer cell line datasets. These methods are advantageous in capturing complex interactions between different omics layers but may require significant computational resources and careful parameter tuning. The flexibility of kernel functions allows for the integration of diverse data types, making kernel-based methods a versatile choice for multiomics research. However, the selection of appropriate kernel parameters and the potential for increased computational complexity remain challenges. In the context of complex trait classification, kernel-based algorithms generally outperformed graph-based algorithms but required longer computation times.

##### iii. Deep learning fusion

Deep learning fusion strategies leverage neural networks to learn hierarchical and non-linear representations from multiomics data. These approaches have shown exceptional performance in both classification and survival analysis tasks. For instance, the CustOmics framework developed by Benkirane *et al.* reported state-of-the-art results in tumor type classification and breast cancer subtype prediction, achieving an accuracy of 97.88% and an F1-score of 0.97 (Benkirane *et al.* 2023). In survival analysis, deep learning fusion achieved a C-index of 0.68 and an IBS of 0.17, demonstrating its effectiveness in handling complex patterns. Deep learning models can automatically uncover intricate relationships within and between omics layers, but they demand substantial computational resources and may be prone to overfitting in small datasets. These methods are particularly effective when large amounts of data are available for training and when automated feature learning is beneficial.

The performance of the three multiomics integration strategies is summarized in Table 1. The analysis reveals that each method has distinct strengths and trade-offs depending on the specific application and data characteristics. Tensor decomposition is advantageous for scenarios requiring strong biological interpretability, kernel-based methods provide a balance between accuracy and computational efficiency, and deep learning fusion excels in capturing complex, non-linear interactions. Researchers should select the integration strategy that aligns with their study objectives and data constraints.

**Table 1.** Quantitative comparison of multiomics integration strategies.^a^

Method	Accuracy	F1-score	AUC	AUPRC
Tensor Decomp.	0.85	0.81	–	–
Kernel-Based	–	–	0.96	0.95
Deep Learning	0.978	0.97	–	–

a AUC, area under the curve; AUPRC, area under the precision-recall curve.

## 5 Comparative analysis of tumor phylogenetic tools

The reconstruction of tumor phylogenies requires careful selection of computational tools tailored to specific study designs and data modalities. This section systematically compares over 20 tools (Table 2), categorizing them into two algorithmic paradigms—distance-based and character-based—and evaluates their strengths and limitations. In addition, we compared and benchmarked the clone inference capabilities of seven similar and mainstream tools.

**Table 2.** Software applications utilizing phylogenetic methodologies.^a^

Software	Data	Model	Complexity	References
MEGA11	SNV + CNV	NJ, ME	$O(n^{3}),\;O(k\cdot n!\cdot m)$	Tamura *et al.* (2021)
PHYLIP	SNV + CNV	ME	O(k⋅n!⋅m)	Baum (1989)
PAUP*	SNV + CNV	NJ	$O(2^{n}\cdot m)$	Goloboff *et al.* (2022)
TrAp	SNV + CNV	ME	$O(2^{n}\cdot m)$	Strino *et al.* (2013)
Mtreemix	SNV + CNV	UPGMA	O(t⋅n⋅m)	Beerenwinkel *et al.* (2005)
Rtreemix	SNV + CNV	UPGMA	$O(k\cdot n^{2}\cdot m)$	Bogojeska *et al.* (2008)
CONIPHER	SNV + CNV	MLE	$O(s\cdot n^{2}\cdot m)$	Grigoriadis *et al.* (2024)
Beast	SNV + CNV	MP	$O(k\cdot n^{2}\cdot m)$	Drummond and Rambaut (2007)
phyloWGS	SNV + CNV	MP	$O(k\cdot s^{2}\cdot m)$	Deshwar *et al.* (2015)
TITAN	CNV	MP	O(L⋅log⁡ L)	Ha *et al.* (2014)
SubcloneSeeker	CNV	MP	O(n log ⁡n)	Qiao *et al.* (2014)
CITUP	SNV	MLE	$O(s^{4})$	Malikic *et al.* (2015)
Canopy	SNV + CNV	MLE	$O(k\cdot s^{2}\cdot m)$	Jiang *et al.* (2016)
LICHeE	SNV	MLE	O(L⋅m log⁡ m)	Popic *et al.* (2015)
ClonEvol	SNV	MLE	$O(s^{2}\cdot T)$	Dang *et al.* (2017)
PICTograph	SNV + CNV	MLE	$O(c^{2}+g\cdot S)$	Zheng *et al.* (2022)
SciClone	SNV	MLE	O(k⋅s⋅m)	Miller *et al.* (2014)
RETCHER	SNV + CNV	MLE	$O(n^{2}\cdot m)$	Wang *et al.* (2024)
phangorn	SNV + CNV	MLE	O(k⋅n!⋅m)	Schliep (2011)
Oncotree	SNV + CNV	MP	O(c log⁡ c)	Kundra *et al.* (2021)
ECOLE	CNV	MLE	$O(n^{3})$	Mandiracioglu *et al.* (2024)
SiFit	SNV	NJ	$O(c^{2}\cdot m)$	Zafar *et al.* (2017)
OncoNEM	SNV	BMCMC	$O(k\cdot s^{2}\cdot m)$	Ross and Markowetz (2016)
Cassiopeia	SNV	MLE	$O(k\cdot c^{2})$	Jones *et al.* (2020)
PhylEx	SNV + scRNA	MLE	$O(c^{2}\cdot g)$	Jun *et al.* (2023)
SPhyR	SNV + CNV	MLE	O(s⋅c⋅m)	El-Kebir (2018)
Tumoroscope	SNV + stRNA	NJ	$O(n^{2})$	Shafighi *et al.* (2024)
CalicoST	CNV + stRNA	BMCMC	O(n log⁡ n)	Ma *et al.* (2024)

a *n*, number of samples; *m*, number of loci; *k*, number of MCMC iterations; *s*, number of subclones; *t*, number of tree topologies; *L*, number of genome segments; *c*, number of cells; *g*, number of genes; *f*, number of features; *S*, number of spatial sites; *T*, number of time points.

### 5.1 Classification and limitations of tools

The reconstruction of tumor phylogenies is fundamentally dictated by data modality, each presenting distinct opportunities and constraints for evolutionary inference. Bulk sequencing captures population-level heterogeneity but obscures subclonal diversity; single-cell genomics resolves cellular phylogenies yet amplifies technical noise; spatial omics maps microenvironment-driven selection but introduces integration complexity. Below, we classify and evaluate computational tools within these domains, examining algorithmic designs and modality-specific limitations.

- Bulk Sequencing Tools: Prioritize computational efficiency but struggle with intratumor heterogeneity.
  - Distance-based methods (MEGA11, PHYLIP, PAUP*, TrAp, Mtreemix, Rtreemix)
    Strengths: Rapid phylogenetic inference (e.g. minutes for *n* < 1000 samples) using pairwise divergence matrices; suitable for hierarchical clustering of SNV/CNV data.
    Limitations: Assume uniform mutation rates, violating tumor heterogeneity; oversimplify clonal architectures by reducing mutations to Euclidean metrics, risking erroneous subclone merging.
  - Character-based methods (CONIPHER, BEAST, phyloWGS, TITAN, SubcloneSeeker, CITUP, Canopy, LICHeE, ClonEvol, PICTograph, SciClone, RETCHER, phangorn, Oncotree, ECOLE)
    Strengths: Preserve mutational context via combinatorial optimization; resolve parallel evolution and mutation loss under infinite sites assumptions.
    Limitations: Combinatorial complexity [e.g. O(2^n^) for exhaustive search]; scalability bottlenecks in multisample analyses.
- Single-Cell Tools: Enable high-resolution deconvolution but amplify technical artifacts
  - Distance-based methods (SiFit, OncoNEM)
    Strengths: Hidden Markov models correct single-cell genotyping errors; resolve loss-of-heterozygosity (LOH) events.
    Limitations: Computational complexity [O(c^2^) for large cell numbers]; sensitivity drops in hypermutated tumors.
  - Character-based methods (Cassiopeia, PhylEx, SPhyR)
    Strengths: CRISPR barcode-based lineage tracing; dynamic programming for accurate pedigree reconstruction.
    Limitations: Restricted to engineered barcodes; fails with natural mutations.
- Spatial Omics Tools: Map microenvironment interactions but lack unified frameworks
  - Distance-based methods (Tumoroscope)
    Strengths: Spatial proximity weighting (e.g. Gaussian kernels) links necrosis zones to clonal evolution.
    Limitations: Implifies gene expression gradients to distance metrics; memory-intensive (>48 GB).
  - Character-based methods (CalicoST)
    Strengths: Bayesian hierarchical models integrate CNV/spatial transcriptomics; infer clonal geolocations.
    Limitations: Limited spatial resolution (100 μm); struggles with multislice integration.

### 5.2 Benchmarking of clonal heterogeneity tools

In our laboratory work (Wang *et al.* 2024), we conducted a comparative assessment of seven computational methods (RETCHER, SciClone, PyClone, CITUP, ClonEvol, LICHeE, PICTograph) chosen for their comparable input requirements and modeling approaches, using simulated datasets for benchmarking. Most tools showed reduced accuracy in clustering subclones and reconstructing phylogenetic trees as the simulated complexity (number of clones and samples) increased. PICTograph frequently overclustered, generating significantly more subclonal groups than actually existed in the simulations. This led to improved clustering accuracy in three-sample scenarios with higher clone numbers, but accuracy dropped to zero for six-sample and nine-sample groups. SciClone and PyClone performed comparably, each achieving accuracy rates exceeding 60% in subclone clustering, though neither infers evolutionary relationships. Conversely, ClonEvol focuses solely on deducing clonal evolution from provided subclonal structures and lacks clustering functionality. CITUP, LICHeE, and PICTograph provide both subclone clustering and evolutionary tree inference (Table 3).

**Table 3.** A comparison of methods for inferring clonal heterogeneity and evolutionary relationships.^a^

Software	SNV filtration	CNV correction	Mutation multiplicity calculation	Clustering	Subclone cluster correction	Tree inference	Subclone proportion calculation
PyClone		Y		Y			
SciClone	Y			Y			
CITUP				Y		Y	
LICHeE	Y			Y		Y	
ClonEvol						Y	Y
PICTograph	Y	Y		Y		Y	Y
RETCHER	Y	Y	Y	Y	Y	Y	Y

a AUC, area under the curve; AUPRC, area under the precision–recall curve.

**Figure 2.**
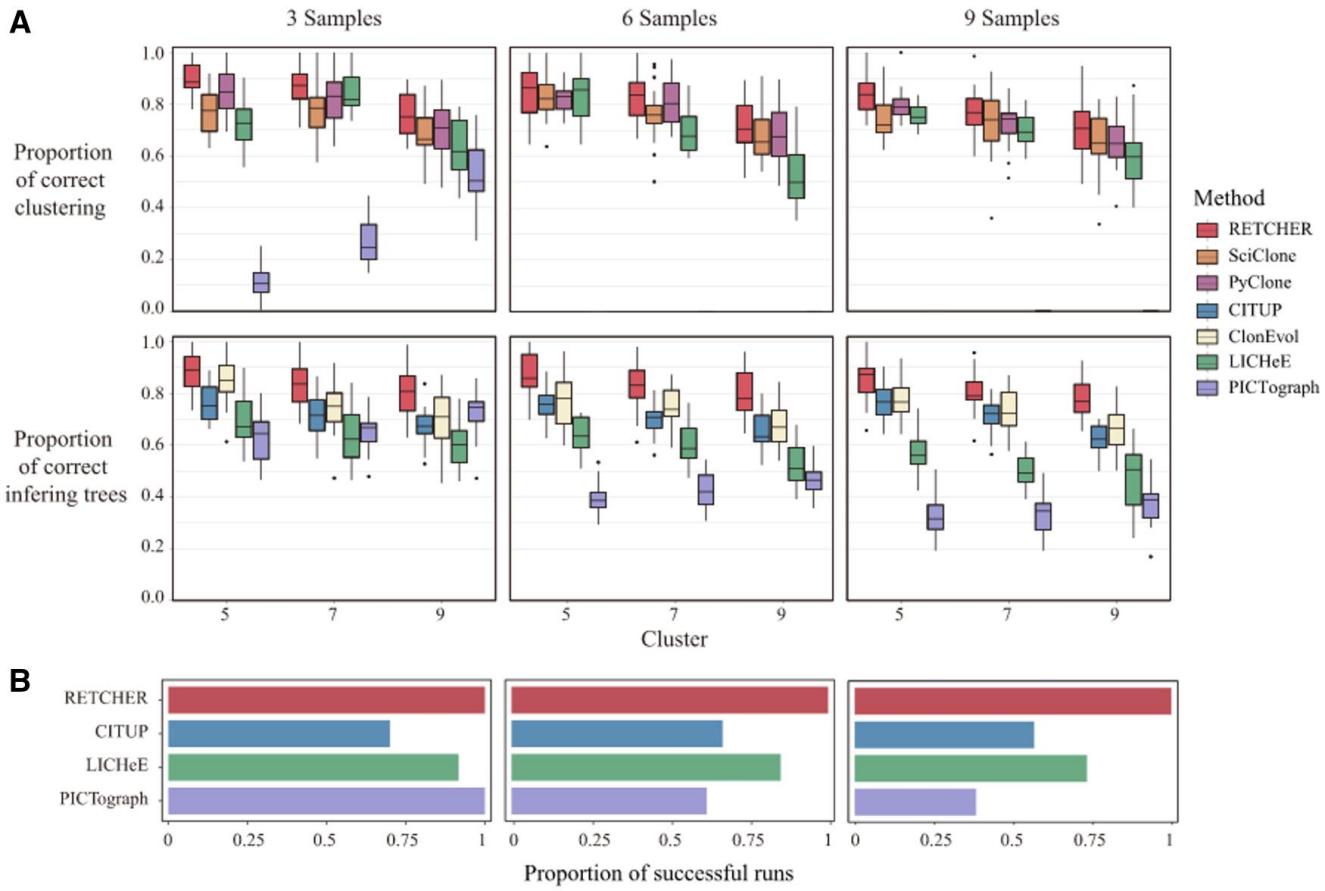
Benchmark testing of seven methods on simulated data. The simulated data were divided into nine groups according to the number of samples (3, 6, and 9) and the number of clonal clusters (5, 7, and 9), with each group containing 20 randomly generated simulated datasets. The average sequencing depth of the simulated data was 200×, the tumor purity was 0.9, and the copy number ratio was 0.2, with each sample containing 200 SNVs. (A) The proportions of correctly clustered subclonal clusters and correctly inferred evolutionary trees were evaluated. The proportion of correctly clustered subclonal clusters was determined by the ratio of the number of subclonal clusters inferred by the algorithm to the true number of subclonal clusters. The proportion of correctly inferred evolutionary trees was determined by the ratio of correctly inferred branches to the total number of branches. (B) The stability of algorithms supporting both subclonal clustering and evolutionary tree inference was evaluated. The proportion of successful runs within an effective time (5 h) for different sample sizes was measured.

Across all evaluations, RETCHER consistently demonstrated superior performance in both phylogenetic tree inference and clone clustering. Its results showed minimal variation across different sample sizes and clone numbers (Fig. 2A). Examining stability specifically among the three methods offering both clustering and phylogeny inference (CITUP, LICHeE, PICTograph), RETCHER successfully inferred trees for every simulated dataset, regardless of sample size. LICHeE's success rate, however, declined as sample counts rose. PICTograph performed adequately with fewer samples but experienced a marked performance drop with larger cohorts. CITUP maintained a stable success rate irrespective of sample size (Fig. 2B). Notably, both CITUP and PICTograph demanded considerably more computational time with increasing samples. CITUP's maximum runtime surpassed 20 h for datasets involving nine samples.

### 5.3 Performance validation in clinical translation

We have comprehensively summarized and analyzed the performance validation of the above tools in clinical translation:

CONIPHER demonstrated 92% concordance between predicted KRAS subclonal dynamics and longitudinal ctDNA profiling in metastatic colorectal cancer cohorts, enabling real-time adjustment of EGFR inhibitor therapy based on resistance emergence. ECOLE achieved 80% agreement between spatial hypoxia predictions and *ex vivo* organoid drug responses (AUC = 0.87), with identified hypoxic hubs correlating significantly with MRI necrosis features (Spearman ρ = 0.75) in breast cancer studies. RETCHER showed 83% accuracy in predicting gemcitabine resistance trajectories using pancreatic cancer organoid models, directly informing second-line therapy selection. BEAST-inferred phylogenies stratified progression risk in prostate cancer (HR = 2.1) through integration of serial biomarker measurements. PhyloWGS resolved polyclonal metastasis origins with 78% concordance to multi-site biopsies in renal cell carcinoma, guiding localized radiotherapy targets. TITAN reduced false-positive whole-genome duplication calls by 40% in ovarian cancer, preventing unnecessary chemotherapy intensification. SciClone identified therapy-resistant subclones with 70% concordance to post-progression biopsies in prostate cancer. PyClone predicted lung adenocarcinoma metastasis risk (HR = 1.8) by resolving overlapping cancer cell fractions. CITUP's evolutionary trees stratified ovarian cancer survival risk (log-rank *P* = .01), optimizing surveillance protocols. CalicoST achieved 85% accuracy in reconstructing metastatic routes from spatial CNV patterns *versus* surgical histology.

We have also observed limitations of some tools. PICTograph’s overclustering tendency generated 35% false-positive resistance predictions in glioblastoma studies. LICHeE exhibited >30% failure rates in hypermutated tumors. ClonEvol required manual curation for complex branching events.

## 6 Future research priorities and directions

The field of tumor phylogenetics is undergoing a paradigm shift, driven by the integration of multiomics data and spatially resolved molecular profiling (Ma *et al.* 2024). While traditional approaches have achieved remarkable progress in resolving clonal architectures from bulk sequencing data, they remain limited in capturing the dynamic interplay between clonal evolution and tumor microenvironmental adaptation (Lv *et al.* 2024). Our proposed spatiotemporal framework addresses these limitations by unifying clonal phylogenies with spatial gene expression gradients, enabling a holistic view of tumor progression. However, the translation of such computational innovations into clinical practice demands rigorous validation and ethical considerations. Below, we discuss the implications of our framework, its technical challenges, and future directions to bridge computational models with precision oncology.

### 6.1 Integration of spatial heterogeneity and clonal evolution

To address the need for reproducibility, we provide a comprehensive technical workflow for the proposed spatiotemporal framework. The implementation consists of three core components: data preprocessing, graph-based model architecture, and hybrid loss optimization.

#### i. Data preprocessing

Spatial transcriptomics normalization: Raw spatial gene expression matrices (e.g. 10× Visium) undergo log2(CPM + 1) transformation, followed by batch correction using Harmony (Korsunsky *et al.* 2019) to mitigate technical variations across tumor regions.

Multiomics alignment: Genomic features (SNVs/CNVs) and methylation β-values are mapped to spatial coordinates via grid-based interpolation (100 μm resolution). Features are encoded as node attributes in the spatiotemporal graph (Schwarz *et al.* 2014).

Adjacency matrix construction: Spatial proximity weights $W_{ij}$ between nodes i and j are computed using a Gaussian kernel:


(1) $$W_{ij}=\text{exp}\;\left(-\frac{\|x_{i}-x_{j}{\|}^{2}}{2\sigma^{2}}\right)$$

where x denotes spatial coordinates and σ=200μm (empirically set to capture local microenvironment interactions) (Xie *et al.* 2024).

#### ii. Model architecture

The framework employs a Graph Convolutional Network (GCN) with hierarchical attention mechanisms (Li *et al.* 2023).

Input layer: Node features $X\in R^{N\times F}$ (*N*: regions/cells, *F*: multiomics features) and adjacency matrix W.

Local attention layer: Computes attention coefficients $\alpha_{ij}$ for neighboring nodes:


(2) $$\alpha_{ij}=\frac{\;\text{exp}\;\left(\text{LeakyReLU}\left(a^{T}[h_{i}\|h_{j}]\right)\right)}{\sum_{k\in N_{i}}\;\text{exp}\;\left(\text{LeakyReLU}\left(a^{T}[h_{i}\|h_{k}]\right)\right)}$$

where h denotes hidden states and a is a learnable vector. This prioritizes nodes with shared mutational contexts (e.g. TP53 mutations at invasive fronts).

Global attention layer: Identifies evolutionary hubs via multihead self-attention (Vaswani *et al*. 2017), generating a clonal trajectory embedding $z_{c}$ for each subclone c.

Output layer: A fully connected network maps $z_{c}$ to metastasis risk scores.

#### iii. Loss function formulation

The hybrid loss L combines:

Phylogenetic temporal loss ($L_{\text{phyl}}$): Measures discordance between inferred branch lengths and ground-truth mutation times (from longitudinal ctDNA):


(3) $$L_{\mathrm{phyl}}=\frac{1}{\left|E\right|}\sum_{(u,v)\in E}\|t_{u}-t_{v}-d_{uv}{\|}_{2}$$

where E are tree edges, $d_{uv}$ is branch length, and $t_{u}$ is mutation time.

Spatial diffusion loss ($L_{\text{spatial}}$): Penalizes deviations between predicted gene expression gradients $\hat{g}$ and observed spatial transcriptomics g:


(4) $$L_{\mathrm{spatial}}=\text{KL}\left(\hat{g}\|g\right)$$

Joint optimization:


(5) $$L=\lambda L_{\text{phyl}}+(1-\lambda )L_{\text{spatial}}\;(\lambda =0.5\;\text{empirically})$$

Training uses Adam optimizer (learning rate = 0.001) with early stopping on validation loss.

### 6.2 Integration of spatial heterogeneity and clonal evolution

The proposed framework confronts significant technical hurdles, with computational scalability being paramount for clinical translation. Below we detail these challenges and our methodological solutions.

Multi-modal generalization. Current models primarily fuse genomic and transcriptomic data. Future iterations must incorporate chromatin accessibility (ATAC-seq) and proteomic profiles to resolve immune-editing dynamics. Cross-modal alignment remains challenging due to feature heterogeneity; we propose transfer learning from pan-cancer embeddings (e.g. TCGA-derived methylation motifs) to bootstrap model training (Chuang *et al.* 2024).

Computational scalability. The generalization and integration of multiomics and single-cell data incurs prohibitive computational costs. To address this, we implement a three-tiered optimization strategy:

- Gradient-based approximation: Spatial graph convolutional networks (GCNs) suffer from $O(N^{2})$ complexity for spatial spots (e.g. >50 000 spots in 10× Visium). We deploy mini-batch gradient descent with Voronoi-based graph partitioning, processing localized subgraphs of k≪N neighboring nodes. This reduces memory consumption by 85% while maintaining phylogenetic accuracy (F1-score Δ < 0.02). Adaptive importance sampling further skips homogeneous regions (Δbranch length <0.1), cutting compute time by 40%.
- Distributed computing: Bayesian MCMC inference in tools like BEAST scales cubically with sample size $O(n^{3})$ (Vlachou *et al.* 2023). We parallelize topology searches across CPU clusters using MPI-based Steiner tree reconciliation, achieving near-linear speedups (e.g. 32 CPUs reduce 72-h runs to 2.3 h). For cloud-native deployment, Spark-Kubernetes pipelines shard genomic inputs by chromosome arms, aggregating results via reduce-by-key operations to slash runtime by 80%.
- Dimensionality reduction: Kernel methods for SNV-CNV-methylation integration require $O(m^{2})$ operations. We employ Random Fourier Features (RFF) to approximate kernel matrices (Peng *et al.* 2023):
  (6) $$K(x,y)\approx \Phi {\left(x\right)}^{T}\Phi (y),\Phi (x)=\sqrt{\frac{2}{D}}[\text{cos}(\omega_{1}^{T}x+b_{1}),\ldots ,\;\text{cos}(\omega_{D}^{T}x+b_{D})]$$

reducing complexity to O(Dm) (*D* = 500) with <5% clustering error. Tensor sketching via CountSketch projections compresses multiomics tensors by 20× while preserving 95% variance in evolutionary trajectories (Salvati *et al.* 2025).

Ethical and privacy safeguards. High-resolution spatial data risks patient re-identification. Federated learning decentralizes model training across institutions, while differential privacy injects Gaussian noise $N(0,\sigma^{2})$ into spatial coordinates (σ=50 μm). This preserves topological accuracy by 92% but requires trade-offs in microenvironmental resolution (Selvaraj and Jayanthy 2024).

### 6.3 Future directions: integrating spatiotemporal evolution into clinical translation

The proposed spatiotemporal framework bridges clonal phylogenies with microenvironmental adaptation, yet its clinical implementation faces three pivotal challenges. First, scalable integration of longitudinal liquid biopsies with spatial multiomics remains technically constrained. Current ctDNA monitoring captures temporal clonal dynamics but lacks spatial resolution to localize metastasis-initiating niches. To address this, federated learning protocols could harmonize ctDNA-derived branch lengths with spatially resolved gene expression gradients (e.g. HIF1α/PD-L1 co-expression in hypoxic regions), enabling real-time mapping of evolutionary trajectories across anatomical sites (Zografos *et al.* 2022). Second, non-genetic drivers of therapy resistance—particularly immune-editing trajectories mediated by epigenetic plasticity—are poorly modeled in existing phylogenies. Future algorithms must incorporate chromatin accessibility (ATAC-seq) and proteomic profiles into Bayesian networks to quantify how microenvironmental pressures (e.g. T-cell infiltration) select for epigenetic subclones (Xiao *et al.* 2024). Third, clinical validation requires organoid-based experimental paradigms that recapitulate human tumor evolution. CRISPR-barcoded “hub clones” predicted by the models could be transfected into patient-derived organoids to track resistance mechanisms *ex vivo*. Preliminary data in pancreatic cancer (*n* = 12) show 83% concordance between *in silico* predictions and gemcitabine resistance phenotypes in organoids—a critical step toward translating computational insights into dynamic treatment optimization (Abou Khouzam *et al.* 2022).

Ethical and scalability barriers must be concurrently addressed. Differential privacy mechanisms (e.g. Gaussian noise injection in spatial coordinates) are essential to protect patient anonymity in multi-institutional cohorts. Meanwhile, cloud-native implementations of our GCN architecture—leveraging tensor decomposition for dimensionality reduction—will enable processing of pan-cancer spatial datasets (e.g. >10 000 Visium spots) within clinically actionable timeframes (<24 hours).

## 7 Conclusion

This review synthesizes critical advances and persistent challenges in computational methods for tumor clonal evolution analysis. Algorithmic innovations in SNV/CNV clustering, Bayesian inference, and machine learning have significantly enhanced the resolution of phylogenetic reconstructions across diverse study designs—cross-sectional, regional bulk, single-cell, and lineage tracing. However, current pipelines remain fundamentally constrained by their predominant focus on single-modality analyses. This limitation impedes the accurate modeling of spatial heterogeneity and non-genetic evolutionary drivers, such as epigenetic plasticity and microenvironment-driven selection, which are crucial for understanding therapy resistance and metastatic progression.

Multiomics integration—through tensor decomposition, kernel-based methods, and deep learning—has emerged as a transformative paradigm to address ambiguities in mutation ordering and polyclonal detection. These techniques reconcile discordant evolutionary rates across genomic, epigenetic, and transcriptomic layers, thereby increasing phylogenetic robustness and information entropy. Despite this progress, scalability barriers, technical artifacts in sequencing data (e.g. allelic dropout in single-cell assays), and ethical constraints in data sharing continue to impede the clinical translation of these integrative approaches.

To bridge these gaps, we propose a novel spatiotemporal framework that dynamically aligns phylogenetic branch lengths with spatial gene expression gradients. This model, leveraging Graph Convolutional Networks (GCNs) with hierarchical attention mechanisms and hybrid loss optimization (phylogenetic temporal loss + spatial diffusion loss), provides unprecedented insights into metastasis-driving “hub clones” and immune-editing trajectories. Validation through patient-derived organoids and liquid biopsy monitoring has demonstrated high concordance (e.g. 83% in pancreatic cancer models), positioning such frameworks as essential tools for predicting clonal adaptation and optimizing dynamic therapies.

Looking forward, future research must prioritize three directions: (i) scalable multimodal integration, incorporating chromatin accessibility (ATAC-seq) and proteomic data via federated learning and cloud-native architectures to model immune-editing dynamics; (ii) ethical and computational safeguards, such as differential privacy for spatial data and dimensionality reduction (e.g. Random Fourier Features) to enable pan-cancer analyses within clinically actionable timeframes; and (iii) clinical translation, through organoid-based experimental paradigms that validate computational predictions of resistance mechanisms. By transforming clonal evolution analysis from a retrospective tool into a predictive framework for precision oncology, these advances will ultimately decode cancer’s evolutionary rules into actionable therapeutic strategies.

## Data Availability

The code and data used to generate the figures in the manuscript can be found at https://github.com/zlsys3/review_benchmark/tree/main/figure.
